# Molecular Characterization of Kunitz-Type Protease Inhibitors from Blister Beetles (Coleoptera, Meloidae)

**DOI:** 10.3390/biom12070988

**Published:** 2022-07-15

**Authors:** Emiliano Fratini, Marianna Nicoletta Rossi, Lucrezia Spagoni, Alessandra Riccieri, Emiliano Mancini, Fabio Polticelli, Marco Alberto Bologna, Paolo Mariottini, Manuela Cervelli

**Affiliations:** 1 Department of Sciences, University of Roma Tre, 00146 Rome, Italy; emiliano.fratini@enea.it (E.F.); mariannanicoletta.rossi@uniroma3.it (M.N.R.); lucrezia.spagoni@uniroma3.it (L.S.); alessandra.riccieri@uniroma3.it (A.R.); fabio.polticelli@uniroma3.it (F.P.); marcoalberto.bologna@uniroma3.it (M.A.B.); paolo.mariottini@uniroma3.it (P.M.); 2 Division of Health Protection Technologies, Italian National Agency for Energy New Technologies and Sustainable Economic Development (ENEA), 00123 Rome, Italy; 3 Department of Biology and Biotechnologies, “Charles Darwin”, Sapienza University, 00185 Rome, Italy; emiliano.mancini@uniroma1.it; 4 Neurodevelopment, Neurogenetics and Molecular Neurobiology Unit, IRCCS Fondazione Santa Lucia, Via del Fosso di Fiorano 64, 00143 Rome, Italy

**Keywords:** Kunitz-type protease inhibitors, transcriptomic analysis, protein modelling, blister beetle

## Abstract

Protease inhibitors are widely studied since the unrestricted activity of proteases can cause extensive organ lesions. In particular, elastase activity is involved in the pathophysiology of acute lung injury, for example during SARS-CoV-2 infection, while serine proteases and thrombin-like proteases are involved in the development and/or pathology of the nervous system. Natural protease inhibitors have the advantage to be reversible and with few side effects and thus are increasingly considered as new drugs. Kunitz-type protease inhibitors (KTPIs), reported in the venom of various organisms, such as wasps, spiders, scorpions, and snakes, have been studied for their potent anticoagulant activity and widespread protease inhibitor activity. Putative KTPI anticoagulants have been identified in transcriptomic resources obtained for two blister beetle species, *Lydus trimaculatus* and *Mylabris variabilis*. The KTPIs of *L. trimaculatus* and *M. variabilis* were characterized by combined transcriptomic and bioinformatics methodologies. The full-length mRNA sequences were divided on the base of the sequence of the active sites of the putative proteins. In silico protein structure analyses of each group of translational products show the biochemical features of the active sites and the potential protease targets. Validation of these genes is the first step for considering these molecules as new drugs for use in medicine.

## 1. Introduction

### 1.1. Proteases’ Functions in Living Organisms

Proteases are a large group of enzymes divided, on the basis of the catalytic site, into metallo-, serine-, cysteine, threonine, and aspartic acid proteases [1]. They play a broad range of actions in all living organisms; thus, their dysregulation is potentially very damaging. For example, thrombin and plasmin are involved in coagulopathies and in bleeding disorders; matrix metalloproteases (MMPs) in inflammation, hypertension, and cancer; and elastase in inflammation and tissue lesions [2,3,4]. In particular, elastase’s unrestrained enzymatic activity leads to symptoms typical of the pathophysiology of acute lung injury [5] that has been described, for example, during severe SARS-CoV-2 infection. Furthermore, the inhibition of neutrophil elastase reduces the symptoms of acute lung damage [6,7] and has been suggested as a potential prophylactic treatment option [8]. Moreover, SARS-CoV-2, as well as many other viruses, uses several different host and viral proteases to complete its viral life cycle.

Growing evidence suggests that members of the serine protease family, including thrombin, chymotrypsin, plasminogen activators, urokinase, and kallikreine, play a role in the development and/or pathology of the nervous system, i.e., serine proteases in neural parenchyma and cerebrospinal fluid after the blood–brain barrier injury and thrombin-like proteases in spinal motor neuron degeneration [9].

### 1.2. Protease Inhibitors as Drugs for Clinical Applications

Considering the multiple roles of proteases, protease inhibitors are considered versatile tools in medicine, agriculture, and biotechnology. Natural or synthetic protease inhibitors have been intensively studied by medical and agricultural researchers, given their important physiological functions. For example, inhibitors of the human protease angiotensin-converting enzyme (ACE) are used in the treatment of cardiovascular disorders [10]. In addition, inhibitors of the HIV protease are widely used in the treatment of HIV infection [11].

Natural protease inhibitors, as opposed to synthetic ones, have the strongest defensive therapeutic roles with few side effects, and it has generally been accepted that reversible inhibitors are preferred over irreversible ones, as the latter are more likely to have toxic side effects [12]. Indeed, numerous examples of natural-derived protease inhibitors have been described so far. For example, the thrombin inhibitor hirudin was initially isolated from the medicinal leech [13], and the *Escherichia coli* protein ecotin has been engineered to create a potent and selective inhibitor of plasma kallikrein [14].

### 1.3. Kunitz-Type Protease Inhibitors (KTPI)

The Kunitz domain is a class of serine protease inhibitors found in many living organisms from animals to microbes. Kunitz-domain inhibitors are classified under the inhibitor family I2, Clan IB according to the MEROPS database [1]. This motif was first identified in the bovine pancreatic trypsin inhibitor (BPTI), which is a strong inhibitor of serine proteases such as trypsin and chymotrypsin. In addition to inhibiting serine proteases, some members of the Kunitz family are also able to inhibit cysteine and aspartic proteases [1]. Kunitz-type protease inhibitors (KTPI), reported in the venom of various organisms, such as cnidarians, wasps, spiders, scorpions, and snakes [15], have been studied for their potent anticoagulant activity and widespread protease inhibitor activity [16]. Moreover, KTPI does not display only inhibitory activity. For example, dendrotoxins, isolated from snakes, block Kv ion channels in neurons, thereby modulating the neuronal activity [17]. The amyloid β-protein (APPI), which accumulates in the neuritic plaques and cerebrovascular deposits of patients with Alzheimer’s disease, also contains a Kunitz-domain sequence [18,19]. Angiopeps, a family of KTPI, are used to deliver pharmacological agents to the brain and Angiopep-2-conjugated molecules showed good tolerance in phase I clinical studies and reached phase II for the treatment of recurrent-breast-cancer brain metastasis [20,21]. As natural protease inhibitors, the Kunitz domain is contained in the protein ecallantide that has recently been approved for the treatment of hereditary angioedema [22]. Derivatives of a Kunitz protein from *Schistosoma mansoni*, identified by RNAseq analysis, have been found very effective in protecting against Schistosomiasis in a mouse model [23].

### 1.4. Kunitz-Type Protease Inhibitors (KTPI) in Insects

In insects, serine protease inhibitors play a central role in the defence against microbial invasion [24,25], for example by inhibiting the germination of conidia and the development of the germ tube of Ascomycetes parasites [26]. The haemolymph coagulation cascade, as occurs in human blood, involves various serine proteases and is strictly regulated by different and specific inhibitors. Similar fine-tuned signalling cascades are present in all insects, but only a few have been studied in detail. In particular, information is very scant or absent in a group of insects in which reflex haemorrhage plays an important evolutionary role. Blister beetles (Coleoptera: Meloidae), a widespread family that includes almost 3000 species [27,28,29,30], if disturbed, naturally exude from the leg joints oily yellowish droplets of haemolymph containing cantharidin (CA), the so-called “reflex-bleeding”. This physiological response has evolved as a defensive strategy against predators, with cantharidin being a toxic terpenoid. Apparently, the release of blood is mediated by an increase in hydrostatic pressure [31]. Easy bleeders exhibit spider-like microstructures on the surface of the cuticles. It is suggested that these microstructures may facilitate fissure of the integument and make it hydrophobic. This last property would allow keeping the exuding haemolymph as a droplet on the integument surface [32], but it should also involve a strict and reversible coagulation control to prevent the loss of too much haemolymph.

### 1.5. KTPI in the Subfamily Meloinae

**Figure 1:**
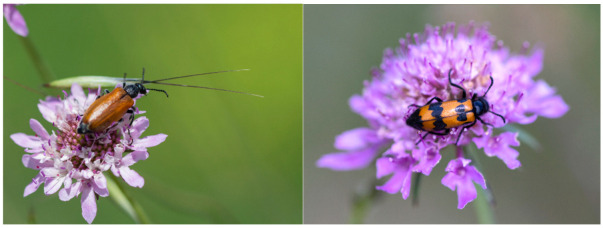
Habitus of the species *Lydus trimaculatus*, (**left**) and *Mylabris variabilis* (**right**).

Putative KTPI anticoagulants can be identified in transcriptomic resources obtained for two blister beetle species, *Lydus trimaculatus* and *Mylabris variabilis* [33]. Both species belong to the subfamily Meloinae. *L. trimaculatus* belongs to the tribe Lyttini [30] and has an East Mediterranean distribution. It is likely a parasitoid of wild bees (Hymenoptera, Apoidea) during its larval stages, while the adults are phytophagous, monophagous, and feed on flowers. *M. variabilis* belongs to the tribe Mylabrini [34] and has a Western Palaearctic distribution. Like all the members of Mylabrini, it is a parasitoid of grasshoppers (Orthoptera, Acridoidea) during its larval stages, whereas the adults are phytophagous, polyphagous, and feed on flowers (Figure 1). The validation of these genes is the first step for considering these molecules as potential new drugs for medical use. In this work, the KTPI of *L. trimaculatus* and *M. variabilis* were characterized by combined transcriptomic and bioinformatics methodologies. On the base of the sequence of the active sites of the putative proteins, transcripts were divided into three sets. In silico protein structure analyses of each group of translational products confirmed their potential biochemical activity on the targeted proteases.

Both species belong to the subfamily Meloinae. *L. trimaculatus* belongs to the tribe Lyttini and *M. variabilis* belongs to the tribe Mylabrini. Adult specimens are depicted feeding on flowers since both species are phytophagous and polyphagous.

## 2. Materials and Methods

### 2.1. BLAST Search for Kunitz-Containing Domain Protein in the Transcriptome of L. trimaculatus and M. variabilis 

The BPTI Kunitz protein (mRNA: NM_001001554.3; Protein: NP_001001554.2) and *Anaplophora glabripennis* Kunitz protein (mRNA: XM_018715645.1; Protein: P_018571161.1) have been utilized by BLAST analyses to retrieve Kunitz protein from the previous transcriptome [33] carried out on the haemolymph, accessory glands, and total bodies of *L. trimaculatus* and *M. variabilis*. The analyses revealed 33 homolog transcripts, among them 15 sequences were selected containing only one Kunitz-type domain. The selected sequences were classified into groups based on their FPKM (fragments per Kb of transcript per million mapped fragments) values: very low expression (bottom 20th percentile; FPKM ≤ 0.06), low expression (bottom 50th percentile; FPKM ≤ 0.12), medium expression (70th percentile; 0.13 < FPKM ≤ 2.62, and 80th percentile; 2.95 < FPKM ≤ 8.59), and high expression (95th percentile; 9.22 < FPKM ≤ 104.62, and 100th percentile > 110.8). Then, we further selected 9 sequences for cloning, discarding the sequences coding for poorly expressed proteins and those that were redundant between the two species.

### 2.2. Molecular Cloning

RNA was extracted from the whole body with Trizol reagent (Invitrogen, Waltham, MA, USA) as performed previously [33]. Total RNA (2 μg), purified from the whole body of *L. trimaculatus* and *M. variabilis*, and primer random hexamers were utilized in 20 μL reaction volume, according to the manufacturer’s procedures (Super-ScriptIII First-Strand Synthesis System for RT-PCR, Invitrogen). A set of specific forward and reverse primers were designed (Table 1) to amplify the selected sequences with Platinum Taq DNA polymerase High Fidelity (Invitrogen) according to the manufacturer’s instructions. RT-PCR conditions were calculated according to the various primer pairs. PCR products were run on 1.8% agarose gel to confirm the molecular weight. Full-length cDNAs were cloned in pCR2.1 vector (The original TA Cloning Kit, Invitrogen) according to the manufacturer’s procedures. Plasmidic DNA was extracted with GenElute Plasmid Miniprep Kit (Sigma, Tokyo, Japan) and 1 μg digested with *Eco*RI (Thermoscientific, Waltham, MA, USA) to assess the correct molecular weight. For each transformation, 5 clones were selected and double-strand sequenced by the Microsynth AG (Balgach, Switzerland).

### 2.3. In Silico Analyses of the Identified Sequences

For each different protein of *L. trimaculatus* and *M. variabilis,* the following analyses were conducted. The cloned cDNAs were analysed using the ExPASy Translate tool (https://web.expasy.org/translate/ (accessed on 11 January 2021 and 10 June 2022) to assign their open reading frames and predict the amino acid sequences’ products. Protein sequences were analysed using SignalP 6.0 (https://services.healthtech.dtu.dk/service.php?SignalP (accessed on 11 January 2021 and 10 June 2022) and Phobius (https://phobius.sbc.su.se/ (accessed on 11 January 2021 and 10 June 2022) servers to predict the presence of signal peptides and the location of their cleavage sites. The analyses of the signal peptides and the Kunitz peptide signature sequences were performed by aligning the sequences with MUSCLE (MUSCLE < multiple sequence alignment < EMBL-EBI) and the alignment outputs were used for sequence logo generation using WEBLOGO [35]. To define the signal peptide and Kunitz signature consensus sequences we used EMBOSScons (EMBOSS Cons < Multiple Sequence Alignment < EMBL-EBI) setting a conservative substitution matrix (BLOSUM80). Representative sequences were chosen, according to a probable consensus, identifying isoforms when possible. For those sequences, BLASTp analyses were carried out to retrieve homologous proteins and conserved domains [36]. In the BLAST analysis, default algorithm parameters and the nonredundant protein sequences database were used. The results obtained were cross-validated using other online resources including InterPro (https://www.ebi.ac.uk/interpro/ (accessed on 15–29 January 2021) and HHpred (https://toolkit.tuebingen.mpg.de/tools/hhpred (accessed on 15–29 January 2021). Among the results, the matches with the best max score and E-value were selected to produce multiple sequence alignments between the target sequences and their respective matches using MUSCLE (http://www.ebi.ac.uk/Tools/msa/muscle/ (accessed on 1 February 2021 and 9 June 2022), yielding an output file in ClustalW format and a pairwise identity matrix [37]. The main aim of the alignment was to identify conserved residues and domains, probably involved in the related function. The structure–function relationships of each group of proteins were deduced by modelling the corresponding 3D structure using SWISS-MODEL (https://swissmodel.expasy.org/ (accessed on 9 September 2021) [38,39,40,41]. Each protein of *L. trimaculatus* and *M. variabilis* was submitted to SWISS-MODEL for homology modelling. Among the templates, those with the best identity and coverage were chosen to build the 3D protein models. Among these, the one showing the highest reliability in both Local Quality and Comparison plots was selected. Finally, the structural models in the PDB format were displayed and analysed with CHIMERA v. 1.11.2, highlighting conserved\functional domains and residues [42]. The superimposition of Kunitz structures wsa obtained using the MatchMaker function of Chimera performing a fit through comparison of similar secondary structure and residues.

## 3. Results

### 3.1. Identification of Translational KTPI Products from Lydus trimaculatus and Mylabris variabilis

**Figure 2:**
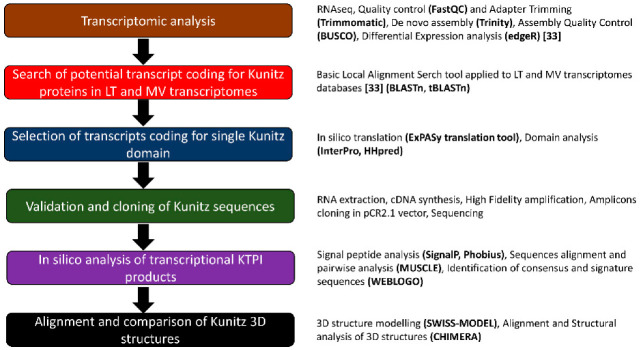
Workflow of the experimental strategy adopted in this study.

**Figure 3:**
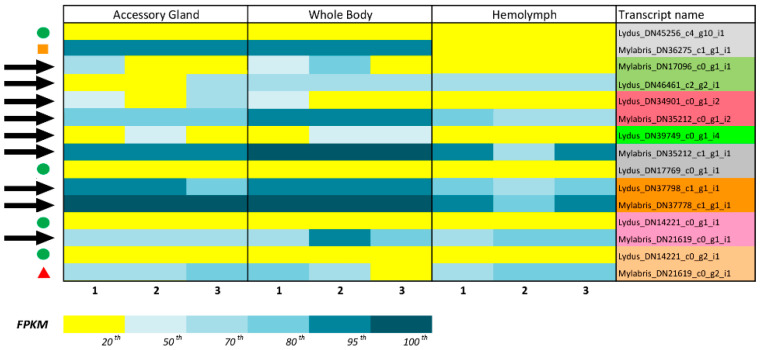
Gene expression for Kunitz transcripts based on RNAseq analysis [33]. Results are visualized as percentile of FPKM; black arrows, transcripts selected for cloning; green circles, transcripts excluded for low expression; orange square, transcript excluded for lack of the initial Met; red triangle, transcript excluded for very high sequence homology with a selected transcript (Mylabris_DN21619_c0_g1_i1 98% homology). Orthologous sequences from *L. trimaculatus* and *M. variabilis* are highlighted with the same colour.

In a previous paper, NGS-based transcriptome analyses of adult samples of *L. trimaculatus* and *M. variabilis* performed by Fratini et al. [33] revealed several proteins involved in the regulation of coagulation from the whole body, accessory gland, and haemolymph. The gene and protein sequences codifying for Bovine Pancreatic Trypsin Inhibitor (BPTI, mRNA: NM_001001554.3; Protein: NP_001001554.2) and Kunitz-Type protease inhibitors of *A. glabripennis* (mRNA: XM_018715645.1; Protein: P_018571161.1) were blasted against the transcriptome of *L. trimaculatus* and *M. variabilis* to identify homologous sequences in these species. A number of 33 transcripts were identified; among them, 15 sequences containing only one Kunitz-Type domain were selected. In Figure 2 the workflow of this study is reported. From those 15 sequences, we selected 9 for cloning (Kunitz_Myl_DN17096, Kunitz_Lyd_DN46461, Kunitz_Lyd_DN34901, Kunitz_Myl_DN35212i2, Kunitz_Lyd_DN39749, Kunitz_Myl_DN35212i1, Kunitz_Lyd_DN37798, Kunitz_Myl_DN37778, Kunitz_Myl_DN21619_g1). We excluded less-expressed sequences (Kunitz_Lyd_DN45256, Kunitz_Lyd_DN17769, Kunitz_Lyd_DN14221_g1, and Kunitz_Lyd_DN14221_g2). Kunitz_Myl_DN21619_g2 was excluded because it was highly homologous (98%) to Kunitz_Myl_DN21619_g1. Kunitz_Myl_DN_36275 was excluded because it was missing the initial Met (Figure 3).

### 3.2. Validation and Cloning of Kunitz Sequences from Lydus trimaculatus and Mylabris variabilis

**Figure 4:**
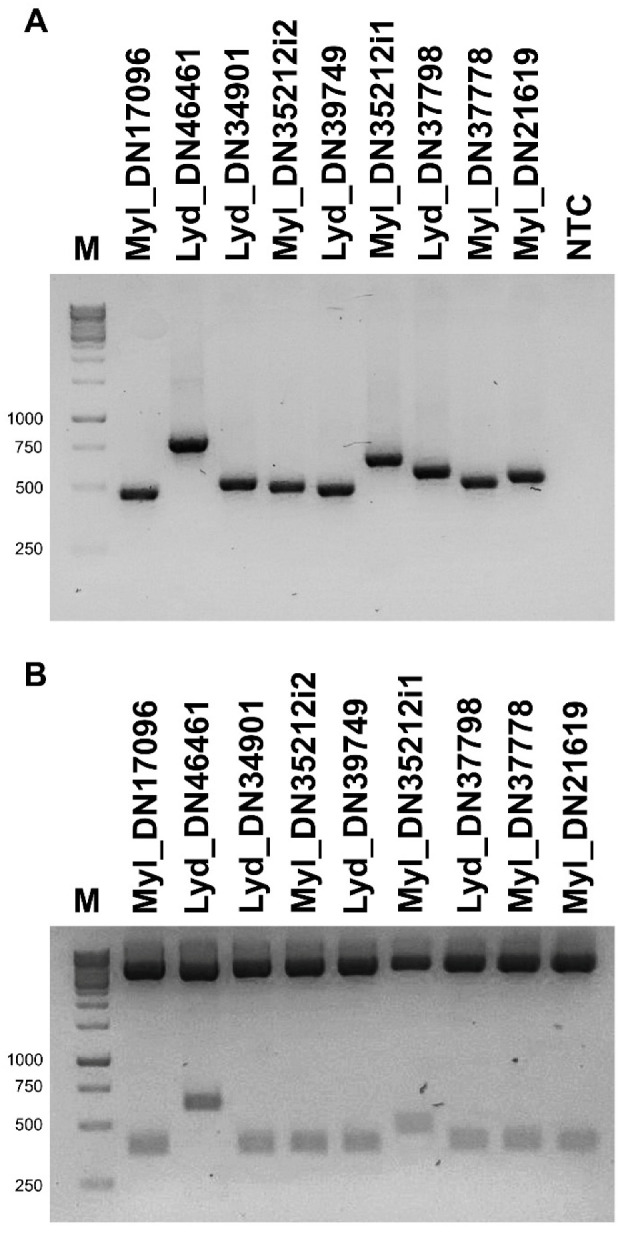
Cloning of nine Kunitz sequences. (**A**) PCR fragments were run on 1.8% agarose gel showing the expected molecular weights. Transcript and protein sequences from sequenced clones. (**B**) pCR2.1 vector containing the 9 Kunitz sequences was digested with *Eco*RI and run on 1.8% agarose gel, showing the expected molecular weights. M, 1 Kb DNA ladder; NTC, negative control.

**Figure 5:**
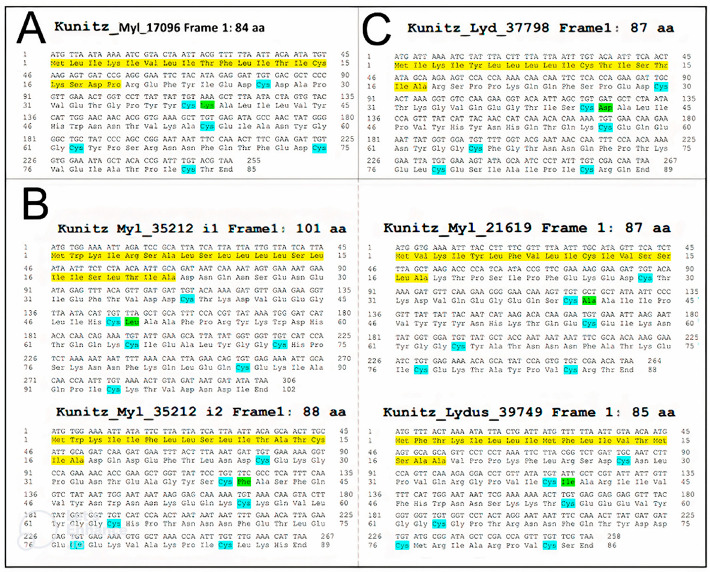
Transcript and protein sequences from sequenced clones. (**A**) Basic P1 residue. (**B**) Nonpolar P1 residues. (**C**) Acidic P1 residue. Yellow, leader sequence; blue, cysteine residues involved in disulphide bridges; green, P1 residue.

Through PCR, using as a template the cDNA retro-transcribed from the total-body RNA of *M. variabilis* and *L. trimaculatus* and primer sets specifically designed for each kunitz-containing transcript (Table 1), nine PCR fragments were isolated, showing the expected molecular weights (Figure 4A). Once amplified, the transcripts were cloned individually within the pCR2.1 vector (Figure 4B) and sequenced to confirm their nature as coding sequences for Kunitz proteins. Then, the sequences obtained were translated with the ExPASy Translate tool [43]. The cDNA cloning confirmed several isoforms of KTPI, with a broad variation in the P1-P1′ sites, as exemplified in Figure 5. The typical residue sequence Lys-Ala in the P1-P1′ position, present in several Kunitz-like serine protease inhibitors such as BPTI (Bovine Pancreatic Trypsin Inhibitor) is present only in the sequence Kun_Myl-17096 of the *M. variabilis* transcript (Figure 5A). Most of the sequences show a hydrophobic side chain at the P1 site: the sequence Kun_Myl-35212_i1 of the *M. variabilis* transcript shows Leu-Ala residues in the P1-P1′ positions, respectively; the sequence Kun_Myl-35212_i2 shows Phe -Ala residues; Ala-Ala residues in the P1-P1′ positions are present in the Kun_Myl-21619 sequence; Ile-Ala residues can be found in the P1-P1′ position of the sequence Kun_Lyd-39749 of the *L. trimaculatus* transcript (Figure 5B and Figure S1). The sequence Kun_Lyd-37798 of the *L. trimaculatus* transcript differs from all the others showing a negatively charged Asp residue in P1 position (Figure 5C).

### 3.3. In Silico Analysis of Translational KTPI Products from Lydus trimaculatus and Mylabris variabilis

**Figure 6:**
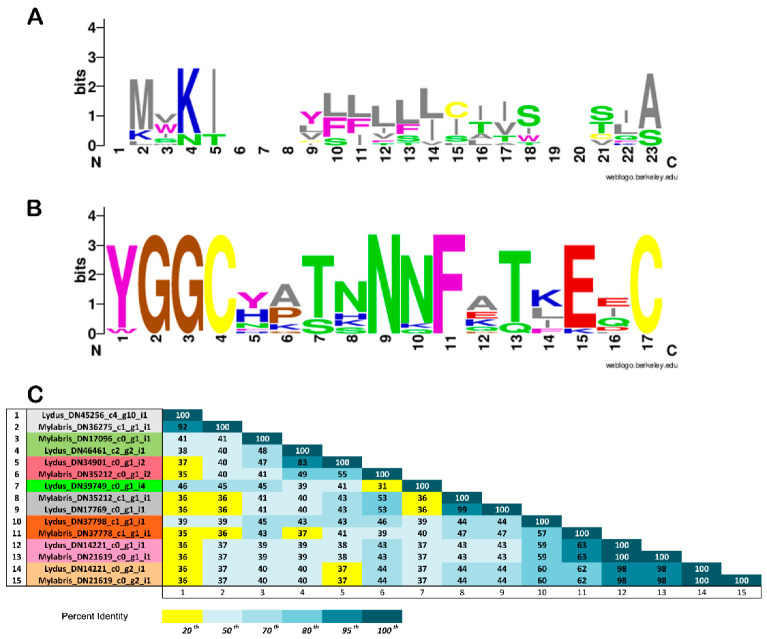
Analyses of the identified translational KTPI products from *Lydus trimaculatus* and *Mylabris variabilis*. (**A**) Leader peptide consensus sequence using the program WEBLOGO. (**B**) Kunitz family signature sequence (amino acids’ colour code: blue, polar positive; red, polar negative; green, polar neutral; grey, nonpolar aliphatic; purple, nonpolar aromatic; brown, proline, glycine; yellow, cysteine. (**C**) Pairwise sequence similarity with percent identity highlighted as percentile. Orthologous sequences from *L. trimaculatus* and *M. variabilis* are highlighted with the same colour of the left-hand column.

**Figure 7:**
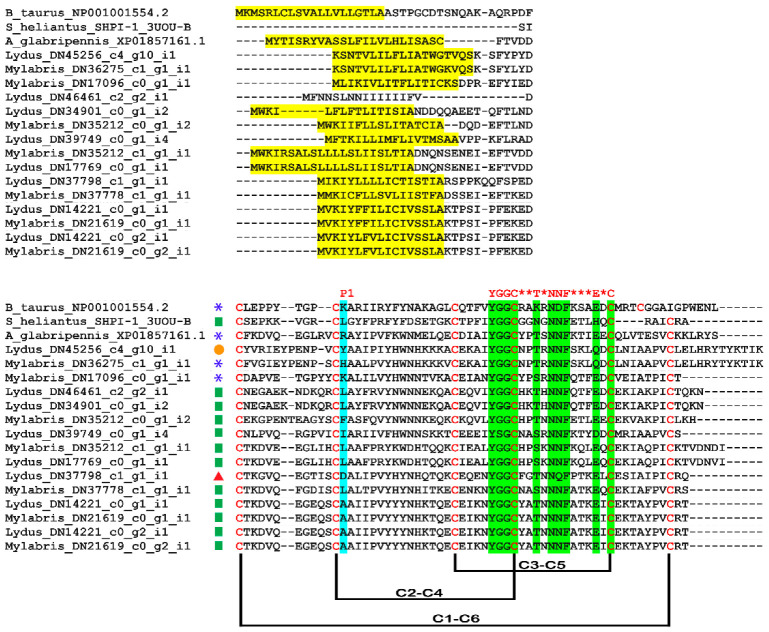
Alignment of Kunitz protein sequences (B_taurus_NP00100554.2, Bovine Pancreatic Trypsin Inhibitor; S_heliantus_SHPI-1_3UOU_B, Chain B, Kunitz-type protease inhibitor SHPI-1; A_glabripennis_XP01857161.1, Kunitz-type serine protease inhibitor 2-like of *Anoplophora glabripennis*. Upper panel: yellow, leader sequence. Lower panel: green, Kunitz family signature sequence; Red asterisks, any aminoacids in the signature sequence; blue, P1 site; blue asterisk, basic P1; green square, hydrophobic P1; orange circle, hydrophilic P1; red triangle, acidic P1; crosslinks, disulphide bridges.

The experimental approach described in the previous section allowed recovering the full-length sequence of 15 plausible mRNA-sequence-encoding proteins characterized by a single KTPI domain, 8 sequences in the transcriptome of *L. trimaculatus,* and 7 sequences in the *M. variabilis* one. Overall, virtually translated KTPI sequences ranged from 85 (*L. trimaculatus* KTPI- DN39749; *M. variabilis* KTPI- DN17096) to 101 (*L. trimaculatus* KTPI-DN17769; *M. variabilis* KTPI-DN35212) amino acids in length and displayed an N-terminal signal peptide. This region, comprising 16–22 residues, displays the consensus sequence MVKIXXXXXLLLLXTISXXTIA, where X is any amino acid, of the leader peptide obtained as described in Materials and Methods (Figure 6A). The overall structure of the Kunitz domain is highly conserved between the two species examined as highlighted in Figure 7 in comparison with the Kunitz domain from the insect *Anoplophora glabripennis* and with the well-characterized Kunitz proteins (BPTI and SHPI-1). The strong conservation between the insect Kunitz domains is shown in Figure S2, derived from a more extensive sequence alignment. All the proteins analysed show the presence of six cysteine residues involved in the characteristic disulphide-bonding pattern of C1–C6, C2–C4, and C3–C5, and the characteristic signature sequence YGGCHXTNNNFXTXEQC (Figure 6B and Figure 7). However, despite the remarkable conservation of the structure, KTPI displayed a highly variable pairwise sequence similarity, ranging from a maximum of 100% identity in the highest-scoring pair (*L. trimaculatus* KTPI- DN14221 vs. *M. variabilis* KTPI- DN21619) to a minimum of 31% (*L. trimaculatus* KTPI-DN39749 vs. *M. variabilis* KTPI-DN35212_c0_g1_i2) (Figure 6C). Moreover, the primary determinant of the specificity for protease inhibition (P1 reactive loop residue) is highly variable, showing different residues possibly capable of inhibiting not only Ser-proteases, but also Cys-proteases (Figure 6). Indeed, for the inhibition of serine proteases, positively charged residues (Lysine and Arginine) have been found at P1 of KTPI-inhibiting trypsin-like enzymes, small hydrophobic residues (Alanine and Valine) in KTPI-inhibiting elastase-like enzymes, and large hydrophobic residues (Phenylalanine, Tyrosine, and Leucine) in KTPI-inhibiting chymotrypsin-like enzymes [44]. Moreover, Leucine residue at the P1 site could also form strong interactions with cysteine proteases such as Cathepsin L [45]. In few cases, acidic residues (Aspartate and Glutamate) have been found at the P1 site of KTPI-inhibiting trypsin [46,47,48]. Finally, there are still few studies on Asp- protease inhibition by KTPI, but it has been proposed that a Lys residue can occupy the P1 site [49,50].

### 3.4. Alignment and Comparison of Kunitz 3D Structures

**Figure 8:**
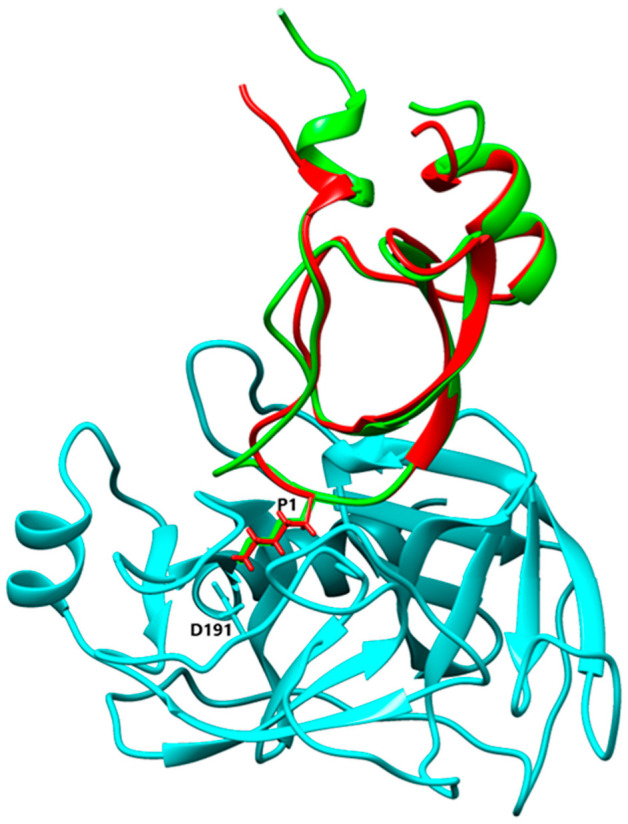
Alignment between Kun_Myl_17096 and Trypsin/BPTI (PBD Code 4Y0Y) [53] structures. Light blue, Trypsin; red, BPTI; green, Kun_Myl_17096. Stick representation has only been shown for Lys20 (P1) and Asp191 residues.

Despite a highly conserved structure with the characteristic disulphide-bonding pattern and signature sequence, the different isoforms of Kunitz from *L. trimaculatus* and *M. variabilis* most probably have different specificity and ability to inhibit different serine proteases due to their high P1-P1′ site variability. The sequences and structures of the nine Kunitz proteins found in *L. trimaculatus* and *M. variabilis* were compared to other Kunitz domains using Swiss-Model [51], and the resulting structural models were aligned to the most similar inhibitor to confirm the potential ability to inhibit the same protease. Kun_Myl_17096 has a Lys residue at the P1 site, and Swiss-Model analysis shows a 41% of sequence homology with BPTI. Aligning the Kun_Myl_17096 structural model with the structure of the Trypsin-BPTI complex (PBD 4Y0Y), the same interactions of the two inhibitors with the catalytic site of Trypsin can be observed (Figure 8). Most Kunitz isoforms of *L. trimaculatus* and *M. variabilis* are characterized by a hydrophobic side chain at the P1 site; in particular, a Leu residue can be found in Kun_Myl-35212_i1, Kun_Myl_37778, Kun_Lyd-46461, and Kun_Lyd-34901. The Kun_Myl-35212_i1 displays 37% sequence similarity with the Kunitz-type Protease Inhibitor (ShPI-1) from the sea anemone *Stichodactyla helianthus* (Hexacorallia and Stichodactylidae). This inhibitor acts on serine peptidases and voltage-gated potassium channels [16,52].

**Figure 9:**
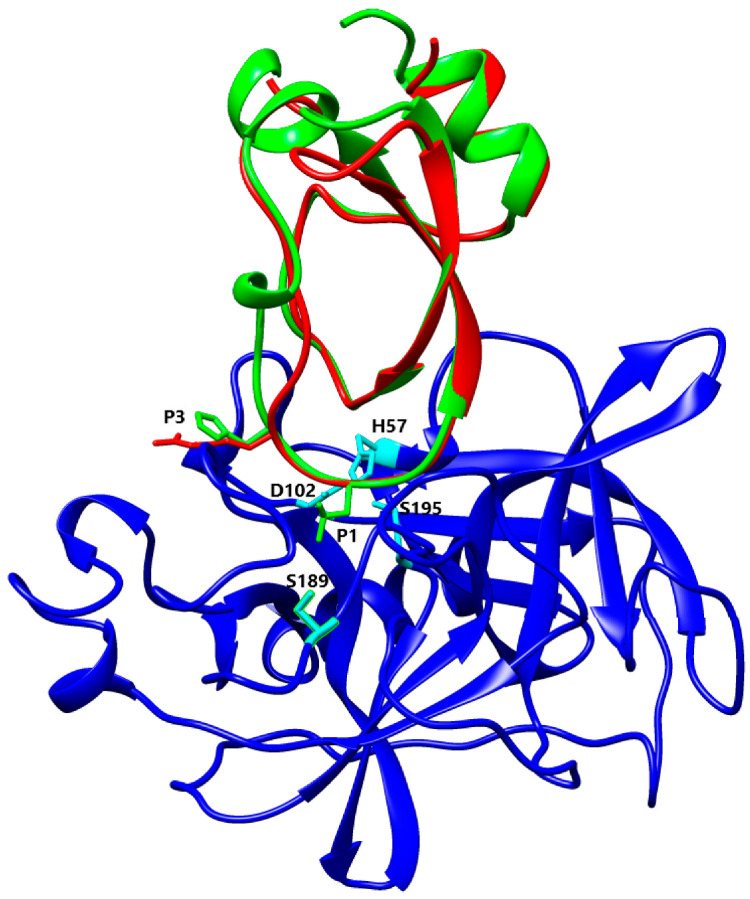
Alignment between Kun_Myl-35212_i1 and ShPI-1 K13L mutant/pancreatic elastase structures (PBD Code 3UOU) [52]. Blue, Elastase with catalytic residues (His57, Asp102, Ser189, and Ser195) in cyan stick representation; red, ShPI-1; green, Kun_Myl-35212_i1 with P1 (Leu13, green) and P3 (Arg11 for ShPI-1 and H11 for Kun_Myl-35212_i1 red and green, respectively) in stick representation.

**Figure 10:**
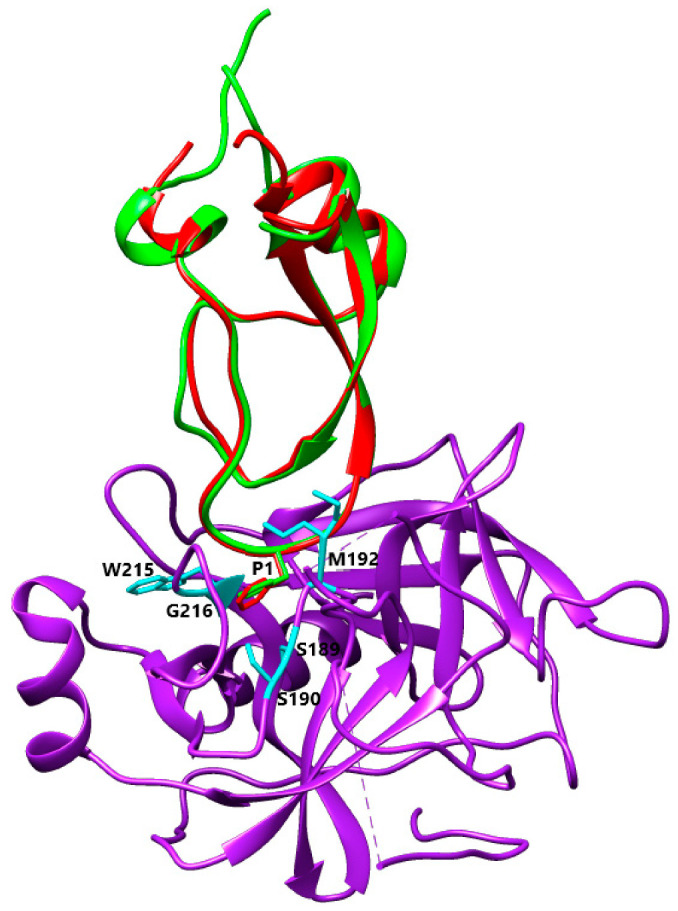
Alignment between Kun_Myl-35212_i2 and BPTI Mutant K15F—Bovine Chymotrypsin Complex (PBD Code 1P2Q) [55]. Purple, Chymotrypsin with S1 pocket walls residues (Ser189, Ser190, Met192, Trp215, and Gly216) in cyan stick representation; red, BPTI Mutant Lys15Phe; green Kun_Myl-35212_i2 with P1 (Phe24) in stick representation.

The superimposition of the Kun_Myl-35212_i1 structural model with the crystal structure of the Kunitz-type protease inhibitor ShPI-1 K13L mutant in the complex with pancreatic elastase evidences the same interacting site for the Kun_Myl-35212_i1 Leu residue (Figure 9). The Kunitz domain with a hydrophobic side chain in the P1 site is believed to be able to interact with chymotrypsin too. The best interaction should be observed with Trp (Ka 5.6 × 10^9^ M^−1^), Tyr (Ka 7.6 × 10^9^ M^−1^), or Phe (Ka 2.5 × 10^9^ M^−1^) residues at the P1 position [54]. The alignment of Kun_Myl-35212_i2, harbouring a Phe residue in P1, with the crystal structure of the P1 Trp BPTI mutant-Bovine Chymotrypsin complex, confirms a similar interaction (Figure 10). The Kun_Lyd-37798 sequence with a negatively charged side chain (Asp residue at the P1 position) corresponds to an unusual Kunitz domain.

Although it displays 41% sequence similarity with the TFPI (Tissue Factor Pathway Inhibitor), it seems far from being a canonical serine protease inhibitor, given the presence of a negatively charged residue at the P1 position.

## 4. Discussion

### 4.1. Meloidae KTPI Identification by Transcriptome Analysis

The transcriptomic analysis conducted on two species of blister beetle [33] allowed broadening the knowledge about the transcripts expressed in *L. trimaculatus* and *M. variabilis*, also opening the possibility to isolate new proteins with potential biotechnological and biomedical functions.

Among these, Kunitz proteins are of particular interest, given their activity as serine, aspartate, and cysteine protease inhibitors [56]. The analysis of transcriptomic data produced an interesting panel of sequences coding for Kunitz proteins. Of the 33 putative sequences displaying a variable degree of homology with the BPTI and Kunitz-Type protease inhibitors of *A. glabripennis*, we decided to focus on 15 sequences codifying for a single Kunitz domain. This selection was made considering that small peptides will be preferable for future biotechnological and pharmacological approaches.

The presence of multiple transcripts in the two species could be possibly due to gene duplication and successive base substitution to give different amino acids at P and P’ sites. This interesting evolutionary aspect could reflect the necessity of insects to widen the panel of protease inhibitors directed at multiple targets. The careful analysis of the sequences allowed us to identify hydrophobic N-terminal signal peptides for all proteins, with the exception of Lydus_DN46461, cleaved during the maturation of the proteins and characteristic of secreted proteins and of many KTPI.

### 4.2. P1 Residue Characteristic and Target Protease Specificities

Despite the remarkable conservation of the structure, KTPI displayed a highly variable pairwise sequence similarity, also in the P1 reactive loop residue, suggesting the possibility to inhibit not only Ser-proteases, but also Asp- and Cys-proteases. In particular, according to the aminoacidic residue in the P1 site, we described three sets of sequences. 

### 4.3. Basic and Nonpolar P1 Residue

The first group, that includes only the Kun_Myl_17096, displays the typical P1-P1′ site Lys-Ala that fills the S1 primary specificity subsite of trypsin(ogen)-like serine (pro)enzymes, forming polar interactions with the invariant negatively charged Asp189 residue [57]. The second group has hydrophobic side chains at the P1 site. The presence of a Leu residue at the P1 site gives a higher affinity for elastases depending on stronger pairwise van der Waals interactions and less unfavourable polar-desolvation drawback compared to inhibitors with Lys residues at P1 [58]. Moreover, the carbonyl oxygen atom of Leu13 occupies the oxyanion hole, establishing three hydrogen bonds with the backbone nitrogen atoms of Elastase residues Gly193, Asp194, and Ser195, together with an H-bond with Ser214 via its nitrogen atom. Additionally, the His11 residue in P3 of Kun_Myl-35212_i1 maintains the same basic properties and position of Arg in S_heliantus_SHPI-1_3UOU_B [52]. As with Leu residue in P1, the large hydrophobic Phe residue is well accepted in both the chymotrypsin and trypsin S1 pockets due to the favourable interactions of the Phe ring with the peptide planes of residues in the pocket walls (191–192 and 215–216), with the Chymotrypsin S1 pocket being more suitable to recognize large hydrophobic residues [55]. Indeed, a Phe residue in P1 has been shown to be correlated to a strong interaction with Chymotrypsin with an association constant value of 2.5 × 10^9^ M^−1^ [59]. These Kunitz-Type proteins could be promising inhibitors of Elastase-like enzymes that are involved in important diseases such as acute pancreatitis, chronic inflammatory lung diseases, and cancer [60,61,62].

### 4.4. Acidic P1 Residue

The third type of Kunitz protein has a negatively charged residue (Asp) in P1. The interaction between the Asp residue and the S1 pocket of serine proteases is strongly unfavourable as suggested by the lowest association constants versus four proteases (Bovine Chymotrypsin, Trypsin, Humane neutrophil elastase, and Salmon Anionic Trypsin) compared to other P1 mutations in BPTI [63]. Nevertheless, acidic residues (Asp and Glu) were found in a Kunitz plant protein, Sporamine, able to inhibit trypsin activity [47], and mutagenesis site-direct experiments demonstrated that Asp and Glu were critical for the inhibitory function. Accordingly, BPTI mutation with an acidic residue at the P1 residue was able to bind trypsin [48]. This amino acid substitution confers the strongest trypsin inhibition at low pH, suggesting a new mechanism in the control of trypsin-like enzymes [48]. In conclusion, even if a good correlation between the P1 residues and the specificity of the target proteases was found, it has to be noticed that the P1 site alone cannot drive protease specificity and that many other aspects need to be considered such as the three-dimensional structure of the protein and of its target, the pH of the solution, the presence of other residues that can modify the context of the P1 residue, and the binding orientation of the P1 residue. In fact, biochemical investigations of a KTPI of *F. hepatica*, FhKT1.3, with an Arg at the P1 position, showed the ability of this protein to inhibit cysteine protease as well as serine proteases. This result could be explained by the binding orientation of Arg residue in the two proteases’ active sites, mediated by the hydrophobic portion of the Arg side chain in the first case or by the guanidine polar group in the second case [45]. We are aware that our data derive from in silico modelling and, even if rigorous, need to be verified by biochemical studies with purified proteins. Nevertheless, they will drive future studies to identify new pharmacological targets. Moreover, it will be interesting to analyse, in the KTPI from *L. trimaculatus* and *M. variabilis,* the occurrence of post-translational modifications as glycosylation, phosphorylation, or presence of pyroglutamate that can modify their biochemical and pharmacological features.

### 4.5. Natural-KTPI Pharmacological Application

The need for finding new protease inhibitors, more effective if taken from natural sources, arises from their growing use in the prevention of adverse effects of acute respiratory distress syndrome, and in the treatment of coagulopathies [8,64,65,66]. The search for effective protease inhibitors has been conducted for at least 50 years and indeed, several of them are already commercialized [2,67]. The interest in using the protein repertoire of Meloidae for this therapeutic purpose is corroborated by a large amount of evidence indicating that protease inhibitors of natural origin, even if with some limitation [67,68], have a greater therapeutic potential (with fewer side effects) than synthetic ones. Furthermore, for the development of therapeutically useful serine protease inhibitors with good pharmacological characteristics, it is generally accepted that reversible inhibitors are preferred to irreversible ones [12].

## 5. Conclusions

In conclusion, the in silico and bioinformatics analyses conducted in this study highlighted the presence of new potential Kunitz-Type proteins derived from Meloidae insects. Even if the data obtained in the present study will need to be confirmed by more extensive studies, we believe our findings could be of interest for future pharmacological applications. Moreover, the description of potential KTPI in two species of Blister beetles increases our knowledge on a group of insects in which reflex haemorrhage plays an important evolutionary role. Thus, this study improves our comprehension of their ecology and adaptation mechanisms. Finally, the study of KTPI from insects expands the understanding on the evolution of this heterogeneous and widespread class of proteins.

## Figures and Tables

**Table 1:** Table list of the primers used for each specific gene.

Transcript	Primers	Primer Name	Fragment Length
Kunitz_Myl_DN17096	5′-TAATAAGAGTTGAACCCCAGC-3′	Kunitz_Myl_DN17096 for	302 bp
	5′-ATCGATCAAAGTACAAATTGCG-3′	Kunitz_Myl_DN17096 rev	
Kunitz_Lyd_DN46461	5′-CTCATCGGTGTATATAAACATC-3′	Kunitz_Lyd_DN46461 for	633 bp
	5′-TTGAGAAAGTTTATTTAATTTTTTTG-3′	Kunitz_Lyd_DN46461 rev	
Kunitz_Lyd_DN34901	5′-CTCATCGGTGTATATAAACATC-3′	Kunitz_Lyd_DN34901 for	380 bp
	5′-TTGAGAAAGTTTATTTAATTTTTTTG-3′	Kunitz_Lyd_DN34901 rev	
Kunitz_Myl_DN35212i2	5′-AAAAGTTGCTTATAAGTAACCAAA-3′	Kunitz_Myl_DN35212i2 for	324 bp
	5′-AACAATTTGGTAAGTTTTTATTATG-3′	Kunitz_Myl_DN35212i2 rev	
Kunitz_Lyd_DN39749	5′-TAATTACACAGCAATAATGTTTAC-3′	Kunitz_Lyd_DN39749 for	301 bp
	5′-GTACTCTACTTTGCTTACCAAAA-3′	Kunitz_Lyd_DN39749 rev	
Kunitz_Myl_DN35212i1	5′-AATCGTAATTATTGTTGTGTATTG-3′	Kunitz_Myl_DN35212i1 for	469 bp
	5′-GACAATTGGTGGGTTATAGTTG-3′	Kunitz_Myl_DN35212i1 rev	
Kunitz_Lyd_DN37798	5′-CATCATAAGATTTTTACATATTGC-3′	Kunitz_Lyd_DN37798 for	320 bp
	5′-GTGAAAATTCAAAATTCCCTCAA-3′	Kunitz_Lyd_DN37798 rev	
Kunitz_ Myl_DN37778	5′-ATTCTAATATCAACAACAATAGCA-3′	Kunitz_Myl_DN37778 for	319 bp
	5′-GTAAAATTTGAATTTATCAATGCTA-3′	Kunitz_Myl_DN37778 rev	
Kunitz_Myl_DN21619	5′-TAATAACACAGCAATAATGTTTAC-3′	Kunitz_Myl_DN21619 for	301 bp
	5′-TTTTGGTAAGCAAAGTAGAGTAC-3′	Kunitz_Myl_DN21619 rev	

## Data Availability

Not applicable.

## Supplementary Materials

The following supporting information can be downloaded at: https://www.mdpi.com/article/10.3390/biom12070988/s1, Figure S1: Transcript and protein sequence from three sequenced clones with Leu at P1 site; Figure S2: Alignment of Kunitz domain sequences from insects.
