# Supplementary material for: Molecular Characterization of Kunitz-Type Protease Inhibitors from Blister Beetles (Coleoptera, Meloidae)
Source: Biomolecules. 2022 Jul 15;12(7):988. doi: 10.3390/biom12070988 (PMC9312475; doi:10.3390/biom12070988)
Supplement: Supplementary file 1 [file biomolecules-12-00988-s001.zip › biomolecules-1762991-supplementary.pdf]

Supplementary Materials

Kunitz_Lyd_34901 Frame 1: 93 aa

```
1     ATG TGG AAA ATT CTA TTC TTA TTT ACA TTA ATT ACA ATT TCT ATT   45
1     Met Trp Lys Ile Leu Phe Leu Phe Thr Leu Ile Thr Ile Ser Ile   15

46    GCA AAT GAT GAT CAA CAA GCT GAA GAA ACA CAA TTT ACA CTA AAT   90
16    Ala Asn Asp Asp Gln Gln Ala Glu Glu Thr Gln Phe Thr Leu Asn   30

91    GAT TGT AAA GAA GGT GCA GAA AAG AAC GAT AAA CAA CGT TGT CTA   135
31    Asp Cys Lys Glu Gly Ala Glu Lys Asn Asp Lys Gln Arg Cys Leu   45

136   GCT TAT TTC CGT GTC TAT AAT TGG AAT AAT GAA AAA CAA GCA TGC   180
46    Ala Tyr Phe Arg Val Tyr Asn Trp Asn Asn Glu Lys Gln Ala Cys   60

181   GAA CAA GTT ATC TAT GGT GGT TGT CAC AAA ACT CAC AAT AAT TTC   225
61    Glu Gln Val Ile Tyr Gly Gly Cys His Lys Thr His Asn Asn Phe   75

226   CAA ACA TTT GAA GAT TGT GAG AAA ATC GCT AAA CCG ATT TGT ACA   270
76    Gln Thr Phe Glu Asp Cys Glu Lys Ile Ala Lys Pro Ile Cys Thr   90

271   CAA AAA AAT TAA   282
91    Gln Lys Asn End   94
```

Kunitz_Myl_37778 Frame 1:87 aa

```
1     ATG ATG AAA ATT TGC TTT CTG TTA TCA GTT TTA ATA ATT TCA ACA   45
1     Met Met Lys Ile Cys Phe Leu Leu Ser Val Leu Ile Ile Ser Thr   15

46    TTC GCT GAT AGT TCA GAA ATA GAA TTC ACC AAA GAA GAT TGT ACT   90
16    Phe Ala Asp Ser Ser Glu Ile Glu Phe Thr Lys Glu Asp Cys Thr   30

91    AAA GAT GTT CAA TTT GGC GAC ATT AGT TGT CTG GCT TTA ACT CCC   135
31    Lys Asp Val Gln Phe Gly Asp Ile Ser Cys Leu Ala Leu Thr Pro   45

136   GTT TAT CAT TAT AAT CAC ATA ACA AAA GAG TGT GAG AAT AAG AAT   180
46    Val Tyr His Tyr Asn His Ile Thr Lys Glu Cys Glu Asn Lys Asn   60

181   TAT GGT GGA TGT AAT GCT AGC AAC AAT AAT TTC GCT ACG AAA GAA   225
61    Tyr Gly Gly Cys Asn Ala Ser Asn Asn Asn Phe Ala Thr Lys Glu   75

226   CAG TGC GAG AAA ATC GCA TTT CCA GTT TGT CGA TCA TAA   264
76    Gln Cys Glu Lys Ile Ala Phe Pro Val Cys Arg Ser End   88
```

Kunitz_Lyd_46461 Frame 1: 79 aa

```
1     ATG TTT AAT AAT AGC TTG AAT AAC ATT ATT ATT ATT ATT ATT TTT   45
1     Met Phe Asn Asn Ser Leu Asn Asn Ile Ile Ile Ile Ile Ile Phe   15

46    GTA GAT TGT AAC GAA GGT GCA GAA AAG AAC GAT AAA CAA CGT TGT   90
16    Val Asp Cys Asn Glu Gly Ala Glu Lys Asn Asp Lys Gln Arg Cys   30

91    CTA GCT TAT TTC CGT GTC TAT AAT TGG AAT AAT GAA AAA CAA GCA   135
31    Leu Ala Tyr Phe Arg Val Tyr Asn Trp Asn Asn Glu Lys Gln Ala   45

136   TGC GAA CAA GTT ATC TAT GGT GGT TGT CAC AAA ACT CAC AAT AAT   180
46    Cys Glu Gln Val Ile Tyr Gly Gly Cys His Lys Thr His Asn Asn   60

181   TTC CAA ACA TTT GAA GAT TGT GAG AAA ATC GCT AAA CCG ATT TGT   225
61    Phe Gln Thr Phe Glu Asp Cys Glu Lys Ile Ala Lys Pro Ile Cys   75

226   ACA CAA AAA AAT TAA   240
76    Thr Gln Lys Asn End   80
```

Figure S1. Transcript and protein sequence from three sequenced clones with Leu at P1 site. Yellow, leader peptide sequence; blue, Cys residues involved in disulphide bridges; green, P1 residue.

Species	Accession	Sequence
Ignelater luminosus	KAF2886195.1	CEMP--FEV-GK----CKMY---YPVWYYNYATDDCERGIYGGCGGNSNRFSHKQDCLD-----HC
Spodoptera frugiperda	XP_035455931.1	CLEE--RQGQGG--ITCFAL---MKRYFYDQTKDKCVSYTYGGCNEGRNSFRNREECEK-----FC
Drosophila serrata	XP_020807810.1	CYDE--PSQTGP----CKAA---FPSWRFIKEKKLCESFLYGGCEATKNIFDDEEECRE-----QC
Athalia rosae	XP_012267160.1	CALP--MER-GY----CRAS---ISRWGYDHQKKTCVKFYYGGCYGNSNNFRTKEECIK-----SC
Ixodes scapularis	XP_029848488.1	CNLP--KDD-GP----CRAM---IPSFYFDNVTRTCHMFMYGGCEGNGNNFESPEQCKE-----EC
Leptotrombidium deliense	RWS25802.1	CLEPPKFEE-QP---RCRAA---ISRWSYNQRTRTCEYFRYGGCNGNHNNFESAEQCMA-----IC
Araneus ventricosus	GBM21980.1	CTEP--RDP-GP----CLGI---FPRWFYNQQTGQCEKFSYGGCFGNKNNFKTKKACES-----IC
Araneus ventricosus	GBL76652.1	CMEP--KDS-GP----CMAL---FPRWFYNQQTGQCEEFTYGGCSGNKNNFKTKNDCEK-----AC
Drosophila innubila	XP_034488093.1	CLQP--VEV-GP----CRAR---IIRWYYNKNTKTCEQFGFGGCHGNQNNFMTLRSCRK-----VC
Drosophila albomicans	XP_034117060.1	CSLP--KDV-GP----CRAR---LVRWYYDRKTKSCEEFDYGGCHGNDNNFKTLRDCRK-----TC
Rhagoletis zephyria	XP_017463616.1	CSQS--LII-GP----CRGM---IPRYYYNADSKSCQGFSYGGCHANGNNFETQEDCED-----KC
Rhipicephalus microplus	XP_037274505.1	CYEA--PET-GR----CRAR---HPSWYFDSRLGHCKMFIYGGCDGNRNRFSTEEECMG-----TC
Digitaria exilis	KAF8763253.1	CVDD--KKT-GP----CRAS---VPSWYYNQETGKCERFTYGGCQGNGNRYSSEEECKS-----NC
Araneus ventricosus	GBM17459.1	CVDD--KET-GP----CRAL---IRSWYYNKETGKCEQFNYGGCKGNGNRYSSEEECKS-----NC
Spodoptera frugiperda	XP_035455487.1	CLDP--IDP-GM----CFAA---YTRYAYDKSSGDCVEFIYGGCKANRNNFSSMDDCRA-----TC
Ephemera danica	KAF4519412.1	CQRP--PAVHGT----CRAD---FSRYSYHSDSNECRRFGYGGCGGNSNNFQTLEACET-----AC
Drosophila erecta	XP_026835025.1	CGQQ--PAKNGDRVITCIGS---FDRYSYHPRIQSCLKFDYGGCNGNDNNFKTLQECEQKCKPENC
Drosophila subobscura	XP_034669849.1	CGQE--PGKQGEVGQTCFAH---FRKYTYYPHMDKCQLFIYGGCGGNDNKFLTMEECEA-----TC
Ooceraea biroi	XP_011337290.1	CSLR--AEA-GL----CKGH---FRKYAWDAVSGKCVEFLYGGCFGNANNFQTKEECEQ-----MC
Ostrinia furnacalis	XP_028178942.1	CTSP--LDS-GP----CLAL---FHVYGYDPDCGECKEFIYGGCHGNRNRYDTLEECQA-ACKSGC
Manduca sexta	XP_037302510.1	CLQE--LKT-GM----CFGY---FPRYGYDPSIKQCKKFVYGGCHGNQNNFATLEECEKRLAQDSI
Manduca sexta	XP_030020428.1	CLQE--LKT-GM----CFGY---FPRYGYDPSIKQCKKFVYGGCHGNQNNFATLEECEK-----TC
Musca domestica	XP_005182680.2	CGLE-HSKSANSQGQSCLAI---IPSWTYNADTKECIYFHYGGCHGNENRFSTKEECLD-----MC
Drosophila innubila	XP_034473706.1	CGEP--HSADGSNGAHCMAY---FPGWSYNADAKECVSFVYGGCGGNNNRFGSKETCEK-----TC
Drosophila obscura	XP_022231023.1	CGLQ--HSRNGDGRISCEAY---IPSWSFDADAKECVKFIYGGCGGNENRFGSKQDCEA-----KC
Trachymyrmex cornetzi	KYN18952.1	CLLP--IKT-GP----CRAD---IQRYAYNSASGQCQQFIYGGCHANANNFKTMSKCEM-----TC
Atta colombica	XP_018055899.1	CLLP--MNT-GP----CRAQ---IPRYAYNSASGQCEQFIYGGCHANANNFKTISECEM-----TC
Atta cephalotes	XP_012054158.1	CLLP--MNT-GP----CRAE---IPRYAYNSASGQCEQFIYGGCHANANNFKTISECEM-----TC
Trachymyrmex cornetzi	XP_018364130.1	CLLP--IKT-GP----CRAD---IQRYAYNSASGQCQQFIYGGCHANANNFKTMSKCEM-----TC
Hermetia illucens	XP_037912489.1	CLEP--QFT-GL----CEAL---FYRYAYNPENKTCEKFVYGGCDANRNNFVTKEDCER-----KC
Nesidiocoris tenuis	CAB0001071.1	CLLP--LKT-GP----CRAD---FPMYGYDVSQKKCVEFQYGGCRGNRNNFKTLEDCQQ-----TC
Nesidiocoris tenuis	CAB0006626.1	CLLP--LKT-GP----CRAD---FPMYGYDVSQKKCVEFQYGGCRGNRNNFKTLEDCQQ-----TC
Bombyx mori	XP_021209234.1	CSLP--IKT-GP----CRGY---FERYAYDSSKHKCVKFIYGGCQQNSNNFETLEKCRA-----AC
Bombyx mandarina	XP_028031339.1	CSLP--AKT-GP----CRGY---FERYAYDSSKHKCVKFIYGGCQQNANNFETLEKCRA-----AC
Bombyx mori	NP_001037044.1	CLLP--IKT-GP----CKGS---FPRYAYDSSEDKCVEFIYGGCQANANNFETIEECEA-----AC
Bombyx mandarina	XP_028031337.1	CLLP--IKT-GP----CRGG---FRRYAYDSSEDKCVEFIYGGCQANANNFETIEECEAACLYNTC
Ceratina calcarata	XP_017879422.1	CLLP--LER-GP----CRAF---IPKYGYNPETNKCELFEYGGCQGNENNFQTLDSCKQ-----VC
Orchesella cincta	ODM94254.1	CQQP--MER-GT----CRAM---HARFYYNSETKKCEKFNYGGCKKNQNNFKTMEECEK-----AC
Teleopsis dalmanni	XP_037959678.1	CYQP--KEV-GR----CFAL---FHRYAYNAENHKCEEFIYGGCAGNKNNFDSLEECEQ-----KC
Drosophila ficusphila	XP_017047325.1	CHQP--IQA-GR----CLAL---FIRFAYNVDTQSCEEFVYGGCGGNQNNFETKDLCEQ-----AC
Drosophila rhopaloa	XP_016970043.1	CHQP--KET-GR----CFAL---FSRYAYNVDTQSCEEFVYGGCAGNKNNFESKEQCEQ-----VC
Drosophila erecta	XP_001968605.1	CHQP--KET-GR----CFAL---FYRYAYNVDTQSCEEFVYGGCAGNKNNFESKEQCEQ-----AC
Drosophila melanogaster	NP_001285595.1	CHQP--KET-GR----CFAL---FYRYAYNVDTQSCEEFVYGGCAGNKNNFESKEQCEQ-----AC
Drosophila simulans	XP_016023333.1	CHQP--KET-GR----CFAL---FYRYAYNVDTQSCEEFVYGGCAGNKNNFESKEQCEQ-----AC
Manduca sexta	XP_030020380.1	CFLP--LEV-GV----CRAL---FFRYGYDPAIKACKEFMYGGCQGNANNFKTLEECQE-----AC
Samia ricini	BAS31066.1	--SP--RAEFGT--RLCRAY---FPSYGYNPSTKECESFTYGGCGGNSNRFNTKPECEE-----AC
Drosophila bipectinata	XP_017097910.1	CAEEFAVKG-GP----CRGL---IPSWSYNQATGKCEPFNYNGCNGSNNRFDNQEDCEK-----NC
Bombyx mori	NP_001119717.2	CEQA--FGNSGP----CFAY---IKLYSYNQKTKKCEEFIYGGCKGNDNRFDTLAECEQ-----KC
Bombyx mori	AAK52496.1	CEQA--FGNSGP----CFAY---IKLYSYNQKTKKCEEFIYGGCKGNDNRFDTLAECEQ-----KC
Bombyx mori	AAN76278.1	CEQA--FGNSGP----CFAY---IKLYSYNQKTKKCEEFIYGGCKGNDNRFDTLAECEQ-----KC
Glossina fuscipes	XP_037883141.1	CSLP--KEI-GP----CRAS---YLKFYYNSGTKACNEFFYGGCQGNANRFDTKEECEN-----LC
Anastrepha obliqua	AUF41106.1	CSQP--REI-GP----CRKA---DPKFFYNSETKACEDFLYGGCKGNDNRFDTKEECEK-----TC
Rhagoletis pomonella	XP_036328511.1	CSQP--QEV-GP----CRKA---DPKFSYNSEKKACEQFFYGGCQGNDNRFDSKEECEK-----NS
Rhagoletis zephyria	XP_017477436.1	CSQP--QEV-GP----CRKA---DPKFSYNSEKKACEQFFYGGCQGNDNRFDTKEECEK-----IC
Aethina tumida	XP_019869895.1	CTKK--AEQ-EP--VVCRGS---LVAFTWDDSKKECVQTRWSGCYKTRNNFQTLDACKK--TAAIC
Leptinotarsa decemlineata	XP_023028705.1	CGLP--VELPGPHGELCLAE---ISVFTWSNLEKKCVPDSFGGCHETNNMFRTEEDCEK-IAKPI-
Diabrotica virgifera v	XP_028150050.1	CFRP--VHD-HKKGSGCPST---IVSWRWDMKAESCKLAAYGGCKPSKNLFFSMRECIR-VAQPVC
Diabrotica virgifera v	XP_028150048.1	CFRP--VHG-SPEPYICYAY---IPSWRWDMKSKACKHVIYGGCNKTKNLFFTKAECEK-VAKPVC
Diabrotica virgifera v	XP_028150047.1	CFRP--VHG-SPEPYICYAY---IPSWRWDMKSKACKHVIYGGCNKTKNLFFTKAECEK-VAKPVC
Diabrotica virgifera v	XP_028150045.1	CFRP--VDG-SPEPISCKAF---LLAWRWDMKSKSCKPSAFGGCNKTKNLFFTKAECEA-VAKPVC
Diabrotica virgifera v	XP_028150046.1	CFRP--VDA-SITPVGCYAN---IPTWRWDMKSKACKYVIFGGCNETKNLFFTKAECEE-VAKPVC
Anoplophora glabripennis	XP_018563480.1	CVKD--VHDERK---VCYAR---IPVFKWDDTIKNCVPAIYMGCHPSNNKFHSIDSCQR-IAGRIC
Diabrotica virgifera v	XP_028128643.1	CSDA--VERRSV--ATCLAR---KTVWTWDVLANKCVTAFYTGCNATKNNFVTEAECLR-VAKPVC
Anoplophora glabripennis	XP_018570741.1	CSIP--PVLEGP---TCNAY---FIRYYWDSAQQRCVKFVYGGCRGTINNFESQRSCER-VAGRVC
Callosobruchus maculatus	VEN34017.1	CIQD--VEGFVG----CKVS---IEVYKWSVEENQCVKAIYGGCHSTRNNFATLEECDA-VARPIC
Diabrotica virgifera v	XP_028139916.1	CFRP--VEY-HPNGIACMAL---IPVWRWDVGAQACVRDTYGGCNPTNNNFPTLEECNE-VARPIC
Diabrotica virgifera v	XP_028139917.1	CYRP--VEY-HPNGIACMAL---IPVWRWDVREEACVRDTYGGCNPTNNNFPTLKECNQ-VARPVC
Diabrotica virgifera v	XP_028153491.1	CIAP--VDK-GG----CKAS---IPVWKYDVFTRTCVEVYYGGCNATNNNFRSFEKCIS-VAGRIC
Diabrotica virgifera v	XP_028153492.1	CIAP--VDK-GG----CKDS---IPVWKYDVFTRTCVKVYYGGCNATNNNFRSFEECIN-VAGRIC
Diabrotica virgifera v	XP_028148524.1	CTAS--VSS-GP--IMCMAY---MPVWKYDINAKHCVRAIYGGCLATKNNFKSLEECET-VTKEVC
Diabrotica virgifera v	XP_028148525.1	CLAP--VHS-GP--IVCLAG---IPVWRYDIYSRICVRDIYGGCNATKNNFKSREECEA-VTKAVC
Aethina tumida	XP_019865378.1	CRLG--PTVSSR---QCKGL---FPRYFWNDDTQECEEVIYGGCRPTKNNFLTMEECIS-IAKPVC
Sitophilus oryzae	XP_030759497.1	CDLP--HTEPGP---QCKAR---IPRFWWNNTSKACQGVIYGGCRATKNNFETLEQCIA-IAGLTC
Sitophilus oryzae	XP_030757304.1	CVLP--HET-GP--IRCLAY---IQVYTWNDSSKKCEPVIYGGCNKTGNNFATLVECEK-TAKAVC
Sitophilus oryzae	XP_030757306.1	CNLP--HEE-GP--IHCLAY---IPVFKWDNAKKQCVPAVYGGCRRTNNNFPTLEQCEL-AARSVC
Anoplophora glabripennis	XP_018568971.1	CHQP--LET-NP---YCNEW---QFAYHWNNALLDCERAVYHGCNPTRNNFITLSACKA-QAEPVC
Acalolepta luxuriosa	BAD52444.1	CYLP--LET-NP---YCNEW---QFAYHWNNALMDCERAIYHGCNRTRNNFITLTACKN-QAGPIC
Leptinotarsa decemlineata	XP_023015035.1	CDEP--LETTSD----CDDW---KFAYHWNNQLKGCERAIYHGCNATRNNFITFNDCEK-QAQPIC
Sitophilus zeamais	ABZ80669.1	CASD--FEN---KWVRCVGR-EGTFIYHWNDKLKGCERAIYKGCAPTRNNFITEHDCNT-QAKPIC
Sitophilus oryzae	XP_030758167.1	CASD--FEN---KWVRCVGQ-EGTFIYHWNDKLKGCERAIYKGCAPTRNNFITEHDCNT-QAKPIC
Dendroctonus ponderosae	ENN79982.1	CSQP--HEN---KWVKCASSGNPAFVYHWNNQLKGCERAIFKGCAPTRNNFVTEKDCNK-QAKPVC
Rhynchophorus ferrugineus	KAF7265443.1	CDQP--IES---KWTKCPGT-SPIFAYHWNHKLKGCERAIYKGCGATRNSFVTERDCQN-QAASIC
Rhynchophorus ferrugineus	ATU47277.1	CDQP--IES---KWTKCPGT-SPIFAYHWNHKLKGCERAIYKGCGATRNSFVTERDCQN-QAASIC
Lydus trimaculatus	DN45256_c4_g10_i1	CYVR--IEY--PENPVCYAA---IPIYHWNHKKKACEKAIYGGCNPTRNNFSKLQDCLN-IAAPVC
Mylabris variabilis	DN36275_c1_g1_i1	CFVG--IEY--PENPSCHAA---LPVYHWNHKKKVCEKAIYGGCNPTRNNFSKLQDCLN-IAAPVC
Mylabris variabilis	DN17096_c0_g1_i1	CDAP--VET-GP--YYCKAL---ILVYHWNNTVKACEIANYGGCYPSRNNFQTFEDCVE-IATPIC
Lydus trimaculatus	DN39749_c0_g1_i4	CNLP--VQR-GP--VICIAR---IIVFHWNNSKKTCEEEIYSGCNASRNNFKTYDDCMR-IAAPVC
Lydus trimaculatus	DN17769_c0_g1_i1	CTKD--VEE-GL--IHCLAA---FPRYKWDHTQQKCIEALYGGCHPSKNNFKQLEQCEK-IAQPIC
Mylabris variabilis	DN35212_c1_g1_i1	CTKD--VEE-GL--IHCLAA---FPRYKWDHTQQKCIEALYGGCHPSKNNFKQLEQCEK-IAQPIC
Diabrotica virgifera v	XP_028128651.1	CSLGVHQDD-GP---VCLAY---IPVYRWSGAEQKCVKDVYGGCRPTNNNFNTEQDCIK-TATPVC
Diabrotica virgifera v	XP_028128650.1	CNLG--VET-GF---RCRAR---KPVYRWSNEDNKCVRDFYGGCRATKNNFRSEDDCVK-TATPVC
Diabrotica virgifera v	XP_028128640.1	CGLG--VQQ-QNGLGVCRAR---IPVYKWSNEDKKCVQVFYGGCHATKNNFREEEECVK-IAKPVC
Diabrotica virgifera v	XP_028128641.1	CGLG--VQQ-QNGLGVCRAR---IPVYKWSNEDKKCVQVFYGGCHATKNNFREEEECVK-IAKPVC
Diabrotica virgifera v	XP_028128649.1	CSLGVEQDD-GP---VCYAS---IPVFRWSDADKKCVKDIYGGCRATKNNFNTEQDCIK-TATPVC
Diabrotica virgifera v	XP_028140837.1	CSLG--VEQDGL---VCQAY---IPVYRWSDAEKKCIKDIYGGCRSTKNNFLTEADCVK-TATPVC
Diabrotica virgifera v	XP_028140838.1	CSLG--VEKDGS---TCEAY---IRVYKWSDAEKKCVKAIYGGCNPTKNNFRTKEDCVK-TATPVC
Diabrotica virgifera v	XP_028128642.1	CALD--VEL-GD--ETCRAL---IPVYKWSIEAKKCVDEFYGGCHPSKNNFKTKAECEK-IATPIC
Diabrotica virgifera v	XP_028128647.1	CTLG--VEQYEE--GICRAL---IPVYRWDNTQKKCVDDFYGGCYATKNNFRHKADCEK-IATPIC
Diabrotica virgifera v	XP_028128648.1	CDLG--VEQ--E--SICRAL---HPVYRWDNTQKKCVDDFYGGCYATKNNFRHKADCEK-IATPIC
Diabrotica virgifera v	XP_028128646.1	CSKH--VHT-GF---ACMAF---IRRYKWNQKEQQCTLAIYGGCHPTKNNFQTFEDCQK-VAEPIC
Lydus trimaculatus	DN37798_c1_g1_i1	CTKG--VQE-GT--ISCDAL---IPVYHYNHQTQKCEQENYGGCFGTNNQFPTKELCES-IAIPIC
Mylabris variabilis	DN37778_c1_g1_i1	CTKD--VQF-GD--ISCLAL---TPVYHYNHITKECENKNYGGCNASNNNFATKEQCEK-IAFPVC
Lydus trimaculatus	DN14221_c0_g1_i1	CTKD--VQE-GE--QSCAAI---IPVYYYNHKTQECEIKNYGGCYATNNNFATKEICEK-TAYPVC
Mylabris variabilis	DN21619_c0_g1_i1	CTKD--VQE-GE--QSCAAI---IPVYYYNHKTQECEIKNYGGCYATNNNFATKEICEK-TAYPVC
Lydus trimaculatus	DN14221_c0_g2_i1	CTKD--VQE-GE--QSCAAI---IPVYYYNHKTQECEIKNYGGCYATNNNFATKEICEK-TAYPVC
Mylabris variabilis	DN21619_c0_g2_i1	CTKD--VQE-GE--QSCAAI---IPVYYYNHKTQECEIKNYGGCYATNNNFATKEICEK-TAYPVC
Mylabris variabilis	DN35212_c0_g1_i2	CEKG--PEN-TEAGYSCFAS---FQVYNWNNKEQKCKQVLYGGCHPTNNNFETLEECEK-VAKPIC
Lydus trimaculatus	DN34901_c0_g1_i2	CNEG--AEKNDK--QRCLAY---FRVYNWNNEKQACEQVIYGGCHKTHNNFQTFEDCEK-IAKPIC
Lydus trimaculatus	DN46461_c2_g2_i1	CNEG--AEKNDK--QRCLAY---FRVYNWNNEKQACEQVIYGGCHKTHNNFQTFEDCEK-IAKPIC
Callosobruchus maculatus	VEN51319.1	CQKD--TEE-GR--EHCRAL---IPVYRWNHSTQKCERAFYGGCNKTKNNFQTLKECEE-VAGSLC
Anoplophora glabripennis	XP_018563467.1	CLKD--TQE-GP--ELCEAY---IKVFKWDDDLKKCEPAIYGGCYATSNNFETIEQCQN-IAEPVC
Anoplophora glabripennis	XP_018571161.1	CFKD--VQE-GL--RVCRAY---IPVFKWNMELQECDIAIYGGCYPTSNNFKTIEECQL-VTESVC
Anoplophora glabripennis	XP_018571160.1	CFKD--VHD-GP--IACMAS---FPVFKWYDELQECDTAIYGGCNPTNNKFETIEECQQ-VAEPVC
Anoplophora glabripennis	XP_018563476.1	CHDD--VSGSNI----CDAY---FMVFRWNDTLQKCVSEFYEGCKPTKNNFHTIEQCQK-IAEPVC
Anoplophora glabripennis	XP_018563478.1	CLKD--VAAPNV----CDAY---FTVFKWNNKLQKCESAVYGGCNPTKNNFPTMEECQQ-VAEPIC
		`                *         : :      *    : **    * :     *`

Figure S2. Alignment of Kunitz domain sequences from insects; green, Kunitz family signature sequence; blue, P1 site; red, Cys involved in disulphide bridges. ***** (asterisk) indicates positions which have a single, fully conserved residue; **:** (colon) indicates conservation between groups of strongly similar properties.
